# Infantile Iron Deficiency Affects Brain Development in Monkeys Even After Treatment of Anemia

**DOI:** 10.3389/fnhum.2021.624107

**Published:** 2021-02-24

**Authors:** Roza M. Vlasova, Qian Wang, Auriel Willette, Martin A. Styner, Gabriele R. Lubach, Pamela J. Kling, Michael K. Georgieff, Raghavendra B. Rao, Christopher L. Coe

**Affiliations:** ^1^Department of Psychiatry, The University of North Carolina at Chapel Hill, Chapel Hill, NC, United States; ^2^Department of Food Science and Human Nutrition, Iowa State University, Ames, IA, United States; ^3^Harlow Center for Biological Psychology, University of Wisconsin-Madison, Madison, WI, United States; ^4^Department of Pediatrics, University of Wisconsin-Madison, Madison, WI, United States; ^5^Department of Pediatrics, University of Minnesota, Minneapolis, MN, United States

**Keywords:** anemia, infancy, brain development, neuroimaging, diffusion tensor imaging, gray matter, monkey

## Abstract

A high percent of oxidative energy metabolism is needed to support brain growth during infancy. Unhealthy diets and limited nutrition, as well as other environmental insults, can compromise these essential developmental processes. In particular, iron deficiency anemia (IDA) has been found to undermine both normal brain growth and neurobehavioral development. Even moderate ID may affect neural maturation because when iron is limited, it is prioritized first to red blood cells over the brain. A primate model was used to investigate the neural effects of a transient ID and if deficits would persist after iron treatment. The large size and postnatal growth of the monkey brain makes the findings relevant to the metabolic and iron needs of human infants, and initiating treatment upon diagnosis of anemia reflects clinical practice. Specifically, this analysis determined whether brain maturation would still be compromised at 1 year of age if an anemic infant was treated promptly once diagnosed. The hematology and iron status of 41 infant rhesus monkeys was screened at 2-month intervals. Fifteen became ID; 12 met clinical criteria for anemia and were administered iron dextran and B vitamins for 1–2 months. MRI scans were acquired at 1 year. The volumetric and diffusion tensor imaging (DTI) measures from the ID infants were compared with monkeys who remained continuously iron sufficient (IS). A prior history of ID was associated with smaller total brain volumes, driven primarily by significantly less total gray matter (GM) and smaller GM volumes in several cortical regions. At the macrostructual level, the effect on white matter volumes (WM) was not as overt. However, DTI analyses of WM microstructure indicated two later-maturating anterior tracts were negatively affected. The findings reaffirm the importance of iron for normal brain development. Given that brain differences were still evident even after iron treatment and following recovery of iron-dependent hematological indices, the results highlight the importance of early detection and preemptive supplementation to limit the neural consequences of ID.

## Introduction

Sadly, the optimal development of many children worldwide is still compromised by an inability to obtain sufficient food and to consume a nutritionally complete diet (Petry et al., 2016; Armitage and Moretti, 2019). Beyond the known effects on growth and general health, poor nutrition can worsen the impact of infection and poverty (Abioye et al., 2019). In addition to the importance of macronutrient intake, extensive research has demonstrated a need for specific micronutrients. Iron has been a particular focus of attention because of the high prevalence of ID in pregnant women and young children, and its essential role in energy metabolism (Gupta et al., 2017). Numerous studies have shown that both too little and too much iron can affect cellular functions throughout the body, especially during the vulnerable fetal and infancy periods when developmental needs for iron are high due to rapid growth (Kwik-Uribe et al., 2000; Yu et al., 2011). The potential for ID to affect neural processes is a major pediatric concern because approximately 60% of oxidative energy in growing infants is devoted to brain development (Steiner, 2019). Iron is essential for oxygen transport and delivery, is involved in mitochondrial energy production through its role in electron transport/oxidative phosphorylation, and it is a critical co-factor and catalyst in many synthetic pathways (Bastian et al., 2019).

To ensure an adequate postnatal supply of iron, many mammalian species, including humans, rely on a 2-stage sequence. First, substantial amounts of maternal iron are transferred across the placenta before birth, which is then supplemented postnatally by the enteral iron in breast milk and later by the iron in solid foods after weaning (Georgieff, 2020). During late pregnancy, placental receptors actively bind iron in maternal blood, and thus can partially protect the fetus from maternal anemia, but the mother’s diet and health can still affect the iron present in hemoglobin and ferritin reserves in tissue at term (Lubach and Coe, 2006). Maternal anemia, placental insufficiency, gestational diabetes, and premature birth have all be found to affect the iron level in neonates (O’Brien et al., 2003; Dosch et al., 2016; Georgieff, 2020). In addition, maternal stress during pregnancy can increase the risk for infantile ID in both animals and humans (Coe et al., 2007; Rendina et al., 2018). These findings have contributed to a paradigm shift in prenatal care. Iron supplements are now routinely recommended both for the mother and to ensure sufficient iron transfer to prevent postnatal ID in infants (Beard et al., 2007; Georgieff, 2020).

Manipulating the dietary supply of iron during pregnancy was the approach used to create a naturalistic animal model of infantile ID. Female monkeys were provided with a level of iron that met the recommended nutrient requirements for non-human primates (National Research Council [NRC], 2003), but is not sufficient to entirely fulfill the higher requirements of the gravid female. It simulates a gestation without the recommended iron supplementation, which is common in low-income countries as well as among lower SES families in high-resource countries, who may not have ready access to prenatal care until late gestation (Armitage and Moretti, 2019). We have shown previously that this prenatal diet results in 20–30% of infant monkeys developing a growth-related ID after birth during the nursing phase, before they recover gradually by consuming the iron in solid foods (Coe et al., 2013). This prevalence is comparable to the rates of anemia caused by iron deficiency in many lower income countries (Petry et al., 2016). Because the infantile anemia in this monkey model is due exclusively to diet, and emerges as the infant’s postnatal growth needs exceed reserves, it has been possible to demonstrate specific effects of ID on peripheral physiology as well as an iron-related reduction in energy metabolism in the CNS (Geguchadze et al., 2008; Patton et al., 2012; Sandri et al., 2020). There is also decreased dopamine activity and upregulated norepinephrine release in the CNS of infant monkeys who had been anemic (Coe et al., 2009), which is similar to the decreased brain dopamine induced in rodent models (Pino et al., 2017). The current analysis addressed the additional question of whether treating an anemic infant with iron at the time of diagnosis could prevent sustained effects on the brain when measured later.

Many prior assessments of human infants and children have documented effects of ID on neurodevelopment (Lozoff et al., 2000, 2006; Georgieff, 2017). The deficits in attentional and emotional processes, sensory processing and neuromotor performance suggest the neural effects are widespread (McCann et al., 2020). However, definitively proving that the long-lasting effects are due solely to iron can be challenging in humans because there are often other dietary deficiencies as well as confounding social factors. One functional MRI evaluation of young adults diagnosed earlier to be anemic during infancy indicated there may be long-lasting effects on neural connectivity (Algarin et al., 2017). That conclusion would concur with experimental models demonstrating that ID during early infancy can affect neurogenesis, synaptogenesis, and dendritogenesis in rodent pups and piglets (Wu et al., 2008; Ferreira et al., 2019).

Iron is especially needed for myelin synthesis by oligodendrocytes, leading to the conclusion that axonal myelination may be a sensitive sentinel indicator of the compromised neurodevelopment (Connor and Menzies, 1996; Todorich et al., 2009). For that reason, our neuroimaging analysis included diffusion tensor imaging (DTI), which can detect deficits in microstructural integrity and myelin maturation. Our *a priori* hypothesis was that later-maturing anterior tracts would be more impacted because the growth-related ID in monkeys typically emerges relatively late between 4 and 8 months of age. Experimental models of early ID in young piglets indicated there were additional effects on GM in limbic areas and throughout the cortex (Leyshon et al., 2016). Therefore, structural MRI analyses were used to determine whether the transient anemia that occurs in older infant monkeys at the end of their nursing period would also affect total brain volume and regional GM volumes, even if treated quickly by administering iron.

## Materials and Methods

### Subjects

Forty-one infant rhesus monkeys (*Macaca mulatta*) born to multiparous adult females were evaluated in this study (19 female and 22 male). These monkeys were part of an established breeding colony imported from India over 50 years ago, but then bred at this facility for over 10 generations (Beck et al., 2019). All were from full-term, unassisted vaginal deliveries and nursed by their mothers through 6 months of age. Between 6 and 7 months of age, the older weanlings were rehoused into small social groups with other juveniles until 12 months of age when the neuroimaging scans were acquired. The scan of one anemic monkey was excluded because of poor quality, resulting in a total of 40 for the final analyses of volumetric and DTI data. These husbandry and experimental procedures were approved by the Institutional Animal Care and Use Committee.

### Housing

During the nursing phase, the infants were raised by their mothers in standardized cages and then moved subsequently to identical double cages with 4 juvenile peers, in order to ensure that housing conditions were similar for all animals across the first year of life. Adult females and older juveniles were fed the same diet (5LFD, LabDiet, St. Louis, MO, United States), supplemented three times per week with fruit and vegetables. Filtered tap water was available *ad libitum*, and tested to verify it was lead-free. The ingredient composition of this diet has been published previously, but important details are provided in Supplementary Table 1. The mean iron concentration in the biscuits (225 mg/kg) meets published nutrient requirements for adult monkeys (National Research Council [NRC], 2003), but does not ensure that all pregnant females will transfer equivalently high levels of iron to their infants (Lubach and Coe, 2006; Coe et al., 2013). The photoperiod was set at 16 h light/8 h dark, with lights turned on at 0600 year-round so that all blood samples would be obtained at the same point in the diurnal cycle.

### Infant Assessments

Blood samples (<3 mL) were collected at 2-month intervals across the first year of life in order to evaluate the infants’ hematology and several iron-related indices. Specimens were collected between 0930 and 1100 via femoral venipuncture. For the purposes of this analysis, only the mean corpuscular volume (MCV) and hemoglobin (Hgb) levels are reported, but we have shown previously that both correlate highly with decreases in serum iron, transferrin saturation, the iron-regulator hepcidin, and ferritin levels when an infant is anemic (Coe et al., 2013). There is also a marked increase in zinc protoporphyrin/heme, which reflects a compensatory response of red blood cells (RBC) to low iron. On the same days as the serial blood collection, the infant was weighed and anthropometric measures recorded to assess growth (e.g., head circumference and crown-rump length).

### Iron Treatment

If the ID reached clinical criteria for anemia–MCV below 50 fL and a Hgb below 10 g/dL–the young monkey was administered iron dextran (10 mg, IM) and B vitamins (B1, 50 mg, B2, 2.5 mg, Niacinamide, 50 mg, B6, 5 mg, d-Panthenol, 5 mg, B12, 50 mg, IM) weekly until its hematology returned back to the normal range (MCV > 60 fL; Hgb > 10 g/dL). Twelve of the 15 ID infants received treatment. The mean number of IM injections was 6 (range 4–8 weeks until effect). MCV and Hgb screening was determined as part of a Complete Blood Count panel by a CLIA-certified clinical laboratory (UnityPoint Health Meriter Labs, Madison, WI, United States). By verifying the continuation of normal leukocyte values, we also ensured that any decrease in iron was not due to acute infection or illness.

### Neuroimaging

MRI scans were acquired when the monkeys were 12–14 months of age (mean age = 374+/−14 days). Briefly, the monkeys received a preanesthetic dose of ketamine (10–15 mg/kg IM) for transport to the imaging facility, followed by dexdomitor (0.015 mg/kg IM) to prevent movement during the scan. The plane of anesthesia was monitored with a pulse oximeter, and body temperature maintained during the 1 h scan. Scans were obtained with a GE MR750 3.0T scanner (General Electric Medical, Milwaukee, WI, United States) using a human 8-channel brain array coil at the Waisman Laboratory for Brain Imaging and Behavior (UW-Madison).

T1 weighted images (124 coronal slices) were acquired using an axial Inversion Recovery (IR) prepared fast gradient echo (fGRE) 3D acquisition sequence (GE BRAVO). The parameters were TI = 450 ms, TR = 8.704 ms, TE = 3.664 ms, flip angle = 12°, matrix = 256 × 256, slice thickness = 0.8 mm, and image gap = −0.4 mm, bandwidth = 31.25 kHz, 80 percent field-of-view in phase encoding direction, 2 averages, with a native voxel resolution 0.55 × 0.55 × 0.8 mm. T2 weighted images (156 sagittal slices) were acquired using a sagittal 3D CUBE FSE sequence with TR = 2500 ms, TE = 81.9 ms, flip angle = 90°, matrix = 256 × 256, 90 percent field of view in the phase encoding direction, slice thickness = 0.6 mm, gap = 0 mm, bandwidth = 62.5 kHz, ARC parallel imaging with a factor of 2 acceleration in both phase encoding and slice encoding directions, voxel size 0.6 × 0.6 × 0.6 mm isotropic resolution.

The DTI protocol used the following parameters: TR = 8000 ms, TE = 83.4 ms, FOV = 16.7 mm, matrix = 128 × 128, 2.6 mm slice thickness with 1.3 mm slice overlap (resolution 1.3 mm × 1.3 mm × 2.6 mm), upsampled to a voxel dimension on the scanner of 0.65 mm × 0.65 mm × 1.3 mm for preprocessing and to an isotropic voxel for diffusion model reconstruction at 0.65 × 0.65 × 0.65 mm. ASSET acceleration factor = 2 in the coronal plane orientation. There were 120 unique gradient directions acquired with 10 baseline images at *b* = 0 s/mm^2^, 10 *b* = 300 s/mm^2^, 40 *b* = 1000 s/mm^2^, and 70 *b* = 2000 s/mm^2^.

### Structural Image Processing

T2-weighted images were registered to the T1-weighted images aligned into a common space. After that step, T1w and T2w images were bias-field corrected, and brain masks for skull stripping were created using AutoSeg_3.3.2 (Wang et al., 2014). Following this preprocessing, T1 and T2-weighted images were jointly segmented into GM, WM and cerebrospinal fluid (CSF) using NeosegPipeline_v1.0.8 (Cherel et al., 2015). Subject-specific probabilistic tissue maps were generated from structural multi-atlas templates via deformable registration and then used in an expectation-maximiation tissue segmentation. Lobar parcellation of the tissue segmentation into 24 bilateral lobar brain regions and eight subcortical GM structures was performed using multi-atlas fusion in AutoSeg_3.3.2 (Wang et al., 2014). All segmentation and parcellation results were quality-controlled visually and no major issues were detected. After the brain parcellation, GM and WM volumes were extracted for each lobar region, and analyzed in addition to total brain volume and GM and WM volumes.

### Diffusion MRI Processing

Preprocessing of DWI images included susceptibility-related distortion correction, eddy current and motion correction using topup (Andersson et al., 2003) and eddy_openmp tools (Andersson and Sotiropoulos, 2016). Automatic removal of artifact-rich images was done using DTIPrep (Oguz et al., 2014). A weighted least-square estimation was employed to produce a diffusion tensor image for each subject. Gradients with high *b*-values (*b* = 2,000 s/mm^2^) were not used in tensor model reconstruction, because higher *b*-value images (i.e., 2,000 s/mm^2^) have a lower signal-to-noise ratio than lower *b*-values (i.e., 1,000 s/mm^2^) and this difference cannot be controlled in the tensor model estimation. Diffusion tensor images were estimated for each monkey using weighted least-square measures. In order to enhance robustness, we chose to exclude gradients from the highest *b*-value shell (*b* = 2,000 s/mm^2^) for the tensor model estimation. Higher *b*-value images (*b* = 2,000 s/mm^2^) have lower signal-to-noise ratios than low *b*-value images (*b* = 300 and 1,000 s/mm^2^) as well as more diffusion artifacts.

Manual brain masks were created from the averaged baseline images of five randomly selected scans (*b* = 0 s/mm^2^) and served as multi-atlas templates to create masks for the other subjects using deformable registration (Avants et al., 2014) and majority voting. DTI images were skull stripped and a study-specific atlas built using DTI Atlas Builder (Verde et al., 2014). Fiber tracts created from a previously created DTI atlas from a different cohort of monkeys^1^ were propagated into the study-specific atlas space. These propagated tracts were voxelized and used as seed maps for fiber tractography. Tractography was performed automatically using AutoTract (Prieto et al., 2016), followed by a manual inspection stage to further clean the tract definitions. The process tracts were then mapped back into each subject’s DTI image space using deformation fields calculated during the atlast building and parameterized along their arc length to yield four diffusion properties: fractional anisotropy (FA), axial diffusivity (AD), mean diffusivity (MD), and radial diffusivity (RD) using DTI Atlas Fiber Analyzer (Verde et al., 2014). These processes steps yielded diffusion profiles for each tract of interest.

### Tracts of Interest

A prior cross-sectional analysis of monkey brain development had demonstrated that larger maturational changes in WM occurred in the temporal and occipital lobes, cingulum and corpus callosum between 1 and 5 years of age (Shi et al., 2013). Thus, for the current analysis, 10 tracts of interest were selected in corresponding brain regions: cingulum (CG), uncinate fasciculus (UF), inferior longitudinal fasciculus (ILF), optic tract (Opt) in left and right hemispheres, and genu (GCC) and splenium (SCC) of the corpus callosum.

### Statistical Analysis

Descriptive summary statistics were generated for the study parameters and their distributions evaluated to determine if transformations were needed. The 40 scans were categorized as being acquired from either continuously IS or ID monkeys based on their prior hematological values between 4 and 8 months of age (*N* = 26 and 14, respectively). MCV and Hgb values were compared by repeated measures analysis of variance to confirm the initial difference between IS and ID infants, and then to verify that the hematological values of previously ID infants had returned to the normal range of IS monkeys at the time of MRI scan. Following significant main effects for iron status and time, as well as a significant interaction term, pairwise *post hoc* comparisons were conducted with *t*-tests. The growth and brain parameters of anemic infants that received iron were also compared to the 3 untreated ones with moderate ID, and then all were combined into one group when found to be similar. Because of technical problems with data quality, one scan was excluded from both the volumetric and DTI analyses, resulting in a total of 14 scans for the previously ID monkeys.

Analyses of the volumetric brain data were conducted in three steps. Anemic status, including Hgb, size, age, and sex were considered as categorical variables and evaluated for their contribution to variance in TBV using a mixed linear model with and without interactions (with SPSS 25.0). Optimal independent variables were iron status, sex, and age. Then, analyses were run to evaluate TBV and GM and WM volumes in identical models, using ID as the predictor, and age at scan and sex as covariates. Finally, secondary analyses with the same models examined volumes in parcellated brain regions to assess if the effects were larger in specific regions. One rationale for the exploratory analyses related to sex was because male monkeys tend to be bigger with a larger head circumference. However, when including either TBV or ICV as a correction, it already adjusted for this sex difference in size. Females and males were similarly represented in both the IS and previously ID groups.

For the DTI analysis, a generalized linear model (GLM) was employed to analyze the effect of ID (i.e., binary variable) on WM maturation. Sex, age at time of scan, and weight at birth, as well as the interaction between sex and iron status were also included as shown below:

[FA,AD,MD,RD]=β+0β(Sex)1+β(Age)2+β(BW)3+β(ID)4+β(ID*Sex)5.

The GLMs were tested first in an omnibus model that analyzed all 4 DTI properties jointly, followed by separate *post hoc* testing of each diffusion parameter. Statistical analyses were performed via FADTTSter (Noel et al., 2017), a tool for Functional Analysis of Diffusion Tensor Tracts Statistics (Zhu et al., 2011). The quality of the extracted fiber profiles were further assessed using minimal pairwise correlation with the average profile (see Supplementary Table 3 for number of scans with high quality profiles; some outliers were excluded from the final analysis). Global omnibus *p*-values were corrected for the number of analyzed tracts using Bonferroni corrections (see Supplementary Table 2 for detailed summary of test results). *Post hoc* analyses were performed for each DTI property to detect local effects within tracts that had evinced a significant effect of ID in the global analysis. Local *p*-values (uncorrected for multiple comparisons) were projected onto the fiber tracts for visualization of significant locations using 3D Slicer (version 4.8.1).

## Results

### Hematological Values

**FIGURE 1:**
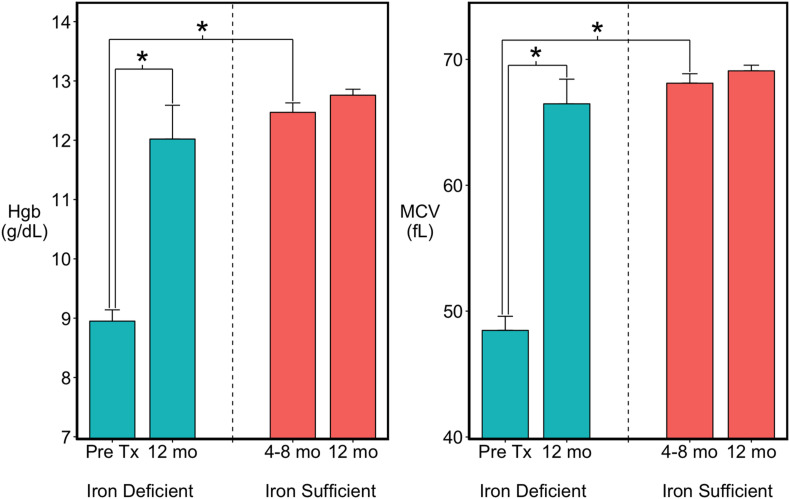
Hematology of the infants categorized as previously iron deficient (ID) on the basis of lowest Hgb and MCV values between 4 and 8 months of age or as continuously iron sufficient (IS). At 1 year of age when MRI scans were acquired, the iron-related hematology of all infants was in the normal range. ^∗^*p* < 0.05.

Based on the occurrence of low MCV and Hgb values between 4 and 8 months of age, 15 of the 41 monkeys were classified as ID (Figure 1). Twelve met clinical criteria for anemia (MCV < 60 fL; HgB < 10 g/dL) and were administered iron dextran IM for a mean of 6 weeks (range 4–8 weeks) until their hematological values returned into the normal range. Three had a moderate ID and were not iron-treated, but were combined with the treated ID monkeys because their growth and brain data were similar. By 1 year of age, when the MRI scans were conducted, the hematological values of all previously ID monkeys, including the untreated ones, did not differ from MCV and Hgb values of monkeys who had remained continuously IS (Figure 1). Similarly, the body weights and head circumferences of IS and ID infants also did not differ significantly at the time of scans [body weight: 2371 (46) vs. 2278 (81) gm; head circumference: 24.6 cm (0.11) vs. 24.4 cm (0.23), IS vs. ID, respectively]. Males tended to be slightly larger than females (153.0 gm, *p* < 0.051) and had a larger head circumference (0.4 cm, *p* < 0.03) at 1 year of age, but both sexes were represented in the IS and previously ID groups. Because of quality issues with one scan, final analyses were conducted with the MRI and DTI data from 26 IS to 14 ID monkeys, excluding 1 iron-treated monkey.

### Physical Growth

Analysis of the body weights at birth and then at 2-month intervals across the first year of life indicated there wasn’t an overall main effect of iron status on overall growth. The birth weights of the IS and ID infants were similar and, as reported above, they were also of similar size at 10 and 12 months prior to acquiring the MRI scans. However, significant 2-way interactions between Age and Iron Status and Sex and Iron Status indicated there were selective differences between IS and ID monkeys at two age points (Supplementary Table 4). At 2 months of age, infants that would become ID were more likely to be larger, reflecting a stronger effect of rapid growth rate on iron utilization in male infants (*p* < 0.03). Conversely, infants that became ID tended to be smaller than IS infants by the end of the anemic period at 6 months of age (IS vs. ID, mean difference = 100 gm, *p* < 0.071). However, by 1 year of age when scanned, the previously ID infants were now of similar size, and there was only the trend for males to be larger than female monkeys (mean difference = 154 gm, *p* < 0.068).

### Structural MRI

**FIGURE 2:**
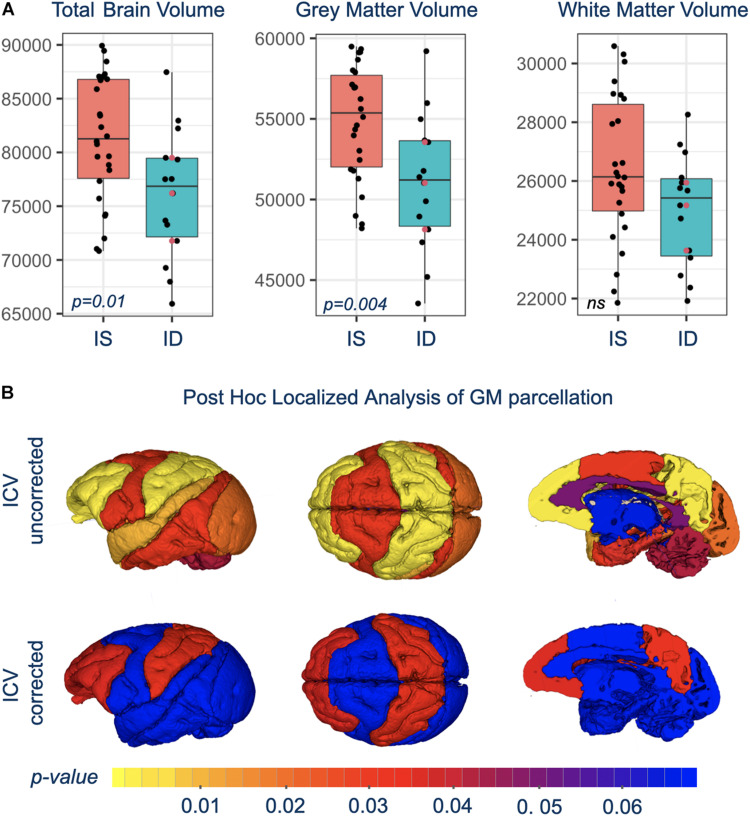
**(A)** Shows box-and-whisker plots with mean difference in brain volumes between IS and previously ID infants. Red circles show the similarity of brain scan results for the untreated, moderate ID monkeys compared to the ID infants treated with iron dextran. **(B)** Is a visual representation of the significance of regional GM differences between IS and previously ID monkeys, without and with correction for ICV.

Total brain volume (TBV) and total GM volumes were significantly smaller in monkeys with a history of ID, and the difference was evident in both untreated and iron-treated monkeys (Figure 2). *Post hoc* analyses of GM volumes in the parcellated regions were conducted to determine if the effect of ID was global or more localized (Table 1). GM volumes in all regions except subcortical structures and cingulate were significantly smaller in monkeys with a prior history of ID. The regional GM values were also analyzed after correction for differences in ICV to further probe if there were larger localized effects in specific regions. This ICV-corrected analysis indicated that smaller GM volumes were most evident in the parietal and prefrontal cortices of the ID monkeys (Table 1). Despite the widespread effects of iron status on GM volumes, there were only statistical trends for global and regional differences in WM volumes at the macrostructural level and they did not reach significance.

**TABLE 1:** Differences in total brain volume (TBV), and gray matter (GM), and white matter (WM) volumes between IS and ID monkeys.

**Region**	**IS mean ± SE**	**ID mean (mm^3^) ± SE**	***p*-value ICV uncorrected**	***p*-value ICV corrected**
**Global analysis**				
TBV	81265 ± 1148	76043 ± 1639	0.01**	**0.030**
GM	54836 ± 697	51046 ± 1145	0.003**	0.008**
WM	26429 ± 484	24997 ± 517	0.069	0.156
***Post hoc* localized analysis of GM after parcellation**				
Occipital	3941 ± 81	3624 ± 105	**0.021**	0.273
Temporal auditory	2073 ± 36	1909 ± 52	**0.007**	0.160
Subcortical	3049 ± 48	2900 ± 71	0.076	0.757
Frontal	3277 ± 50	3087 ± 76	**0.034**	0.959
Cerebellum	2607 ± 50	2437 ± 73	**0.049**	0.476
Insula	374 ± 9	342 ± 14	**0.018**	0.274
Cingulate	979 ± 20	916 ± 25	0.052	0.885
Parietal	3509 ± 51	3220 ± 85	0.002**	**0.026**
Prefrontal	2980 ± 50	2731 ± 64	0.002**	**0.028**
Temporal visual	3066 ± 47	2886 ± 79	**0.032**	0.631
Temporal limbic	1287 ± 19	1215 ± 33	**0.037**	0.588

*Post hoc* contrasts for parcellated tissue volumes are shown both uncorrected and corrected for brain size as reflected by ICV. Significant group differences shown with and without correction for ICV. Bold font indicates *p*-values below 0.05. **p-values that remained significant after Bonferroni correction for number of models in this global analysis. Adjusted for sex and age at scan as covariates.

### Diffusion MRI

**FIGURE 3:**
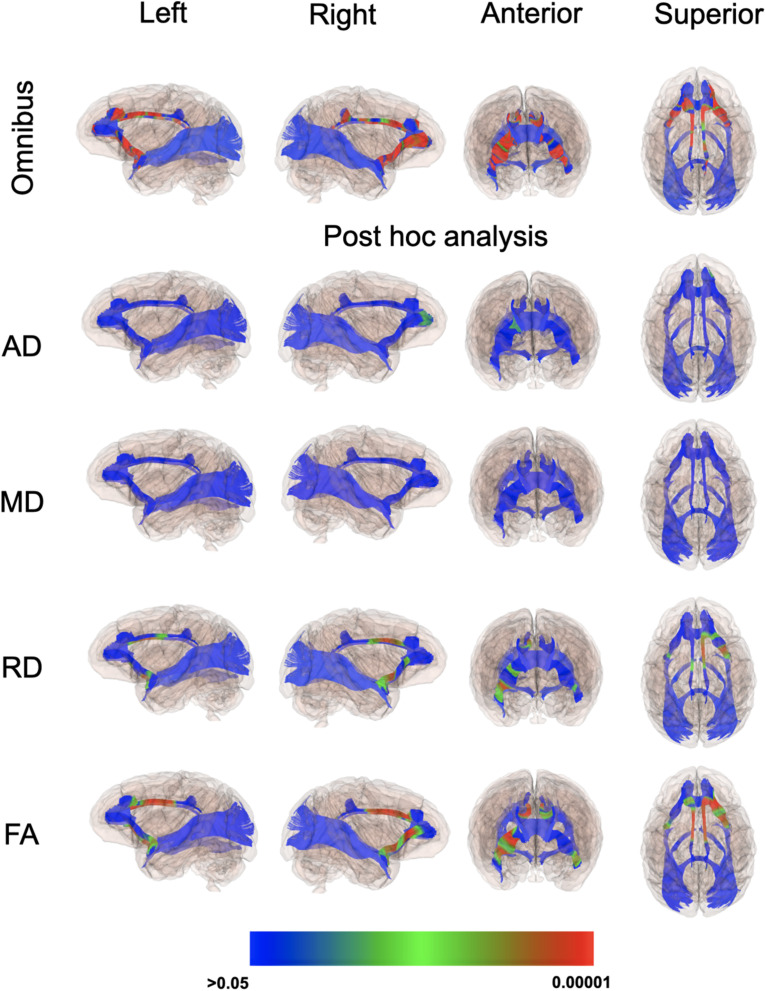
*P*-value color scale illustrating the statistical significance of the local differences between IS and previously ID infants in the global omnibus analysis and separately for each of the four diffusion properties.

**FIGURE 4:**
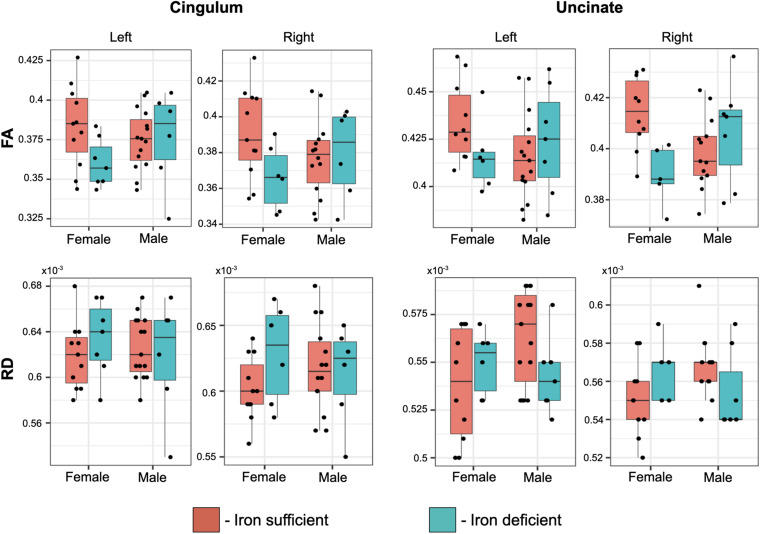
Box plots of the effect of transient ID on FA and RD values in the cingulum and uncinate fascicularis, averaged per tract. The magnitude of the ID effect was significantly larger in females and more evident in the right hemisphere, resulting in a significant interaction between a prior history of ID and the monkey’s sex.

Even though there weren’t overt macrostructural differences in WM, the global omnibus analysis of DTI values indicated the cingulum (CG) and uncinate fasciculus (UF) had been affected by a history of ID. Based on *post hoc* analyses, FA and RD parameters were the largest contributors to this effect of ID in the omnibus analysis (Figure 3). Monkeys in the ID group had lower FA values in left and right CG and UF, and a higher RD in the left and right CG and right UF, indicative of less integrity and myelin content. The differences appeared to be driven more by the female monkeys with a history of ID (Figure 4). Sex-related differences were evident in both hemispheres, and a significant interaction term between sex and magnitude of the ID effect suggested females had been more impacted. In addition, the sex- and ID-related differences were more pronounced in the right hemisphere after Bonferroni corrections for multiple comparisons. Finally, it should be mentioned that ID was not associated with less WM integrity in the two callosal tracts we analyzed (i.e., genu and splenium) or in the posterior tracts, including ILF and Optic tracts.

## Discussion

This analysis has demonstrated that a transient period of ID during infancy can significantly affect brain development in young monkeys. Finding that this effect persisted even after iron treatment, and months after hematological recovery, is in keeping with prior research documenting iron is essential for normal brain growth and that ID can have lasting effects on the developing brain (Georgieff, 2017; Wang et al., 2019). Iron is needed to support neurogenesis, axonal growth, synaptogenesis and dendritic arborization during both the prenatal and postnatal periods. Using this primate model and the same diet, we had shown previously that the growth-related ID that first becomes evident in late infancy can affect oxidative energy metabolism in the periphery and in the CNS (Rao et al., 2018; Sandri et al., 2020). However, it was not known if iron treatment at the time of diagnosing anemia could prevent or lessen the persistence of brain effects. Despite rapid iron treatment, there continued to be a significant impact of ID on total brain size at 1 year of age. In addition to the global reduction in GM volume, focal effects were found in the prefrontal and parietal lobes. The effects on total and regional WM volumes were just statistical trends at the macrostructural level. However, at the microstructural level, there were significant differences in the DTI properties of two tracts, indicative of an effect of ID on myelin integrity in these later-maturing tracts.

Prior research on iron and brain development has highlighted the importance of iron for the synthesis of myelin. Therefore, we anticipated larger effects on WM volumes. But it is important to consider that the maturation of WM in monkeys extends out beyond the selected scan age of 1 year. Increases in WM are sustained through the pubertal transition at 3–4 years. Even when a genetically induced aberration in WM synthesis was investigated in a transgenic monkey model of Huntington’s disease, the progressive deficits in WM became more evident later during the second year of life, with peak differences emerging in the third year (Meng et al., 2017). Both the timing of infantile ID and our scan age preceded this later period of WM maturation. At 1 year, significant effects of the prior ID were detected only with DTI in the uncinate fascicularis and cingulum. Future studies will have to determine if larger effects on WM volumes would be more apparent if the neuroimaging was repeated again at an older age. Alternatively, it is possible that there was sufficient plasticity and enough time for a recovery of myelin synthesis given that the ID lasted for only 2–3 months and resolved by 8 months of age with treatment.

Nevertheless, the persistent effects on GM volumes are still concerning. The brain of an infant monkey at birth is 60% of adult size and with rapid growth will reach 90% by 1 year (Knickmeyer et al., 2010; Scott et al., 2016). Thus, the large effect on GM volumes still present at 1 year could indicate that some of the neural differences will be sustained. That conclusion would be in keeping with previous metabolomic and proteomic analyses of CSF, which indicated the hypometabolic state of the CNS in ID monkeys may both precede and last beyond the period of hematological anemia (Rao et al., 2013, 2018). Similar findings on brain energetics have also been reported in rodent models of ID (Bastian et al., 2010, 2012).

In this monkey model, the emergence of ID first becomes clearly evident when the infant’s growth-related needs for iron deplete prenatally acquired reserves and exceed the iron supply available in breast milk. It is not likely that the observed brain effects at 1 year were induced by limited iron in the fetus prior to birth. Even when pregnant monkeys consume a diet with lower iron levels, most infants typically have high ferritin levels at term, and even the ones that will become ID still have hematological values in the normal range until 2 months of age. However, we should acknowledge the importance of iron even for the fetal brain (Wang et al., 2019; McCann et al., 2020). It has also been shown that the levels of iron reserves at birth can be predictive of the risk for later anemia (Shao et al., 2020). Further, a neuroimaging assessment of human infants born to adolescent mothers who did not take sufficient iron while pregnant and were then scanned 2 weeks after delivery also found more prominent deficits in GM than WM (Monk et al., 2015). Relatively less GM in a rodent model of fetal/neonatal ID was attributed to truncated neurite outgrowth and reduced dendritic arborization (Bastian et al., 2016). Neurogenesis is extremely active during the late fetal stage (Gilmore et al., 2018), so it is possible that even small reductions in iron could have a long-lasting influence on axonal growth and synaptogenesis, which might then contribute to the later differences in GM volumes we found at 1 year.

It may also be of potential relevance that other neuroimaging studies of normal brain development in healthy monkeys without any diet manipulations detected a transient slowing of brain growth at weaning between 6 and 10 months of age (Scott et al., 2016). Thus, the occurrence of ID at this developmental transition to consuming solid food could have accentuated a normal deceleration in the rate of brain growth, which was then prolonged and still evident when scanned at 1 year. The larger effects on GM volume in the parietal lobe could reflect the overall size of this cortical region or a differential vulnerability because the maturing parietal cortex was also found to be particularly sensitive to the effects of maternal infection with influenza virus (Short et al., 2010). Growth of the parietal lobe is sustained postnatally and its connections extend during the developmental period when the ID occurred (Chow and Hutt, 1953; Knickmeyer et al., 2010; Scott et al., 2016; Sawiak et al., 2018).

Diffusion tensor imaging allowed us to look more closely at the microstructure of WM, and revealed bilateral reductions in FA in the cingulum and uncinate fascicularis. In addition, there were higher RD values in the same tracts, but only in the right hemisphere. While FA is sensitive to the developmental pace of myelination, it is not specific to just maturation, and can reflect other processes, including fiber organization. RD has been more specifically linked to axonal myelination, as it reflects perpendicular diffusivity and the myelin sheath restricts diffusion in that direction (Alexander et al., 2007). These myelin differences were observed in ID infants whether they were rapidly treated or allowed to gradually recover via consumption of iron in food. In contrast, similar deficits were not evident in corpus callosal and posterior tracts, which develop earlier, likely reflecting the late onset of ID between 4 and 8 months of age. The observation of some effects on myelination is not a surprising finding. Iron is a required co-factor for lipid biosynthesis, and iron-rich oligodendrocytes are involved in the generation of the myelin sheath around the axon (Connor and Menzies, 1996; Todorich et al., 2009). Moreover, myelin synthesis is an energy-demanding process (Harris and Attwell, 2012), and energy-generating pathways are altered in the CNS of an anemic monkey (Rao et al., 2018).

Many experimental models of ID have focused more specifically on the vulnerability of the hippocampus (Pino et al., 2017). Our imaging results indicate there are additional effects on anterior tracts, which may help to explain some of the previously reported deficits in cognitive functions that rely on inter-hemispheric processing, as well as the effects on attentional and emotional processes. Those evaluations have indicated that some functional limitations persist even after iron therapy. One study found delayed auditory brainstem responses in children still evident at 12- and 18-month follow-ups (Roncagliolo et al., 1998). In animal models of ID, transferring rats from a low iron diet to a iron-sufficient one did not prevent the effect on axons in the optic nerve, including some structural deformation and smaller myelin sheath thickness (DeMaman et al., 2010). Similarly, with inbred mice strains that experienced a genetically programmed and time-delimited period of ID in the hippocampus, persistent transcriptomic and structural abnormalities were found to still be present in the adult hippocampus (Fretham et al., 2012; Barks et al., 2018).

It is important to emphasize that the experimental approach used to create this primate model of ID did not require any additional manipulations postnatally. Fifteen of 41 infants became ID as their growth-related needs for iron exceeded tissue reserves and the iron available in breastmilk. The prevalence of ID in the current study is consistent with previous experiments that found approximately 20–30% became ID by 4–8 months of age (Coe et al., 2013). This percentage is also similar to the prevalence of infants who become anemic in low-income countries due to iron deficiency, after excluding other types of anemia that can be caused by parasitic infections and chronic disease during childhood (Armitage and Moretti, 2019). The path to ID during infancy is known to be multifactorial and includes large maternal weight gains during pregnancy, gestational diabetes, and hypertension, as well as the experience of maternal stress during pregnancy (Coe et al., 2007; Rendina et al., 2018). Collectively, it conveys the broader significance of iron biology for understanding how the effects of early life adversity are first initiated (Phillips et al., 2014).

When considering the translational relevance for public policy and the promotion of maternal and child health, it is important to reiterate that premature birth is still a common occurrence, especially in the context of adversity and poverty. Premature infants are much more likely to be iron deficient (Moreno-Fernandez et al., 2019). In addition, the later growth-related ID seen in the older infant monkeys is similar to what can occur in a subset of breastfed human infants, especially if exclusively breastfeeding past 6–12 months of age. Given that some effects on the brain may begin earlier and persist longer than hematological indices convey, it highlights the need to consider early pediatric screening of infants with known risk factors for anemia, especially after a premature birth (Baker et al., 2010). There may also be benefits of preemptive iron supplementation for a rapidly growing baby who is not fed iron-fortified formula or provided with some solid foods.

## Data Availability Statement

The raw data supporting the conclusions of this article will be made available by the authors, without undue reservation.

## Ethics Statement

The animal study was reviewed and approved by Animal Care and Use Committee of the University of Wisconsin.

## Author Contributions

RV, MS, PK, MG, RR, GL, and CC were involved in the funded project. QW and AW contributed to the analysis of the MRI data and writing. RV and QW worked together to analyze the volumetric and DTI results. All authors have reviewed several manuscript drafts and the final version of the manuscript.

## Conflict of Interest

The authors declare that the research was conducted in the absence of any commercial or financial relationships that could be construed as a potential conflict of interest.

## Footnotes

1 https://www.nitrc.org/projects/unc_macaque/

## Supplementary Material

Supplementary Table 1 Ingredient composition, as well as mineral and vitamin concentrations, for the standard diet fed to the pregnant and nursing mothers and then to the juvenile monkeys after weaning in this study^1^. ^1^LabDiet, 5LFD. ^2^Based on typical daily consumption (200 g) the adult female monkeys were provided approximately 45.0 mg Fe from the biscuit diet each day.

Supplementary Table 2 Diffusion MRI omnibus global *p*-values. Diffusion global omnibus *p-*values for Left and Right Hemisphere (cont.). ^∗^*p-*value remained significant after Bonferroni correction for number of tracts.

Supplementary Table 3 Number of subjects remaining in the analysis after profile QC.

Supplementary Table 4 Body weights (gm) of infant monkeys at 2-month intervals from birth through one year of age when the MRI scans were acquired. Mean values (+SE) shown for males and females, as well as for the monkeys that remained continuously iron sufficient (IS) or became transiently iron deficient (ID). ^∗^Significant main effect of age indicative of normal growth, *p* < 0.001. ^∗∗^Significant main effect of sex, with mean weight for males larger than females, *p* < 0.05. ^‡^Mean weights for IS and ID infants did not differ. However, 2-way interactions between Age and Iron Status, and Sex and Iron Status in the ANOVA followed by *post hoc* testing indicated the weights of IS and ID infants differed at 2 time points. At 2 months of age, male ID infants were the largest (*p* < 0.03), whereas at 6 months the ID monkeys tended to be somewhat smaller after the period of anemia than IS monkeys (*p* < 0.08). ^@^Weights at 10 months and then at 12 months of age when the MRI scans were acquired did not differ between IS and ID. The hematology of the previously ID monkeys was also in the normal range at the time of the scans.
