# Supplementary material for: Infantile Iron Deficiency Affects Brain Development in Monkeys Even After Treatment of Anemia
Source: Front Hum Neurosci. 2021 Feb 24;15:624107. doi: 10.3389/fnhum.2021.624107 (PMC7947927; doi:10.3389/fnhum.2021.624107)
Supplement: Supplementary Table 1 — Ingredient composition, as well as mineral and vitamin concentrations, for the standard diet fed to the pregnant and nursing mothers and then to the juvenile monkeys after weaning in this study1. 1LabDiet, 5LFD. 2Based on typical daily consumption (200 g) the adult female monkeys were provided approximately 45.0 mg Fe from the biscuit diet each day. [file Data_Sheet_1.pdf]

Supplemental Table 1. Ingredient composition, as well as mineral and vitamin concentrations, for the standard diet fed to the pregnant and nursing mothers and then the juvenile monkeys after weaning in this study.[1]

Fe concentration	225 mg/kg
Ingredients	
Protein, %	15.7
Carbohydrate, %	69.1
Fat, %	6.0
Ash, %	5.3
Fiber (crude), %	4.5
Minerals	
Iron[2] , mg/kg	225.0
Zinc, mg/kg	110.0
Copper, mg/kg	21.0
Vitamins	
A, IU/g	20.0
B12, mcg/kg	.073
C, mg/kg	500.0

[1]LabDiet, 5LFD

[2]Based on typical daily consumption (200 g) the adult female monkeys were provided approximately 45.0 mg Fe from the biscuit diet each day

Supplemental Table 2. Diffusion MRI omnibus global p-values

Tracts	Variables	Omnibus p-value	
GCC	Sex	0.203	
	Age	0.566	
	WB	0.797	
	Fe	0.291	
	Fe*Sex	0.028	
SCC	Sex	0.066	
	Age	0.459	
	WB	0.179	
	Fe	0.586	
	Fe*Sex	0.293	
		**Left**	**Right**
CG	Sex	**0.003***	**<0.0001***
	Age	0.011	**<0.0001***
	WB	0.026	0.581
	Fe	**0.001***	**0.001***
	Fe*Sex	0.017	**<0.0001***
UF	Sex	0.035	**<0.0001***
	Age	0.012	**0.003***
	WB	0.002	0.269
	Fe	**0.003***	**0.004***
	Fe*Sex	0.034	**<0.0001***
ILF	Sex	**<0.0001***	0.027
	Age	0.017	0.139
	WB	0.106	**0.003***
	Fe	0.133	0.028
	Fe*Sex	0.725	0.048

Diffusion global omnibus *p* values for Left and Right Hemisphere (cont.)

		Left	Right
Opt	Sex	0.008	0.026
	Age	0.067	0.15
	WB	**<0.0001***	0.007
	Fe	0.044	0.265
	Fe*Sex	0.133	0.194

**p* value remained significant after Bonferroni correction for number of tracts

Supplemental Table 3. Number of subjects remaining in the analysis after the profile QC.

	IS	ID
GCC	20	12
SCC	26	14
CG_L	26	12
CG_R	25	12
UF_L	25	13
UF_R	23	12
ILF_L	26	14
ILF_R	26	14
Opt_L	26	14
Opt_R	26	14

Supplemental Table 4. Body weights (gm) of infant monkeys at 2-month intervals from birth through one year of age when the MRI scans were acquired. Mean values (+SE) shown for males and females, as well as for the monkeys that remained continuously iron sufficient (IS) or became transiently iron deficient (ID).

Age*	Birth	2	4	6	8	1 0	12[@]
Male**							
IS	521 (18)	774 (28)	1173 (32)	1504 (43)	1891 (47)	2131 (151)	2406 (56)
ID[‡]	530 (20)	865 (36)	1145 (24)	1428 (64)	1846 (80)	2107 (91)	2407 (92)
Female**							
IS	512 (16)	795 (22)	1156 (43)	1426 (39)	1819 (58)	2096 (71)	2328 (76)
ID[‡]	480 (25)	740 (39)	1046 (51)	1303 (64)	1656 (88)	1897 (104)	2149 (121)

*Significant main effect of age indicative of normal growth, $p<0.001$
**Significant main effect of sex, with mean weight for males larger than females, $p<.05$
[‡]Mean weights for IS and ID infants did not differ. However, 2-way interactions between Age & Iron Status, and Sex & Iron Status in the ANOVA followed by post hoc testing indicated the weights of IS and ID infants differed at 2 time points. At 2 months of age, male ID infants were the largest ($p<0.03$), whereas at 6 months the ID monkeys tended to be somewhat smaller after the period of anemia than IS monkeys ($p<0.08$).
[@]Weights at 10 months and then at 12 months of age when the MRI scans were acquired did not differ between IS and ID. The hematology of the previously ID monkeys was also in the normal range at the time of the scans
